# Production of poly-γ-glutamic acid by a thermotolerant glutamate-independent strain and comparative analysis of the glutamate dependent difference

**DOI:** 10.1186/s13568-017-0512-0

**Published:** 2017-11-25

**Authors:** Wei Zeng, Guiguang Chen, Ye Guo, Bin Zhang, Mengna Dong, Yange Wu, Jun Wang, Zhiqun Che, Zhiqun Liang

**Affiliations:** 1 State Key Laboratory for Conservation and Utilization of Subtropical Agro-bioresources, Guangxi University, 100 Daxue Road, Nanning, 530004 Guangxi China; 2 College of Life Science and Technology, Guangxi University, 100 Daxue Road, Nanning, 530004 Guangxi China

**Keywords:** Poly-γ-glutamic acid, *Bacillus subtilis* GXG-5, Thermotolerant, Glutamate dependent difference, Genome analysis

## Abstract

Poly-γ-glutamic acid (γ-PGA) is a promising microbial polymer with wide applications in industry, agriculture and medicine. In this study, a novel glutamate-independent γ-PGA producing strain with thermotolerant characteristics was isolated and identified as *Bacillus subtilis* GXG-5, then its product was also characterized. The fermentation process was optimized by single-factor tests, and results showed that high temperature (50 °C) was especially suitable for the γ-PGA production by GXG-5. The γ-PGA yield reached 19.50 ± 0.75 g/L with substrate conversion efficiency of 78% at 50 °C in 10 L fermentor. Comparison of GXG-5 and GXA-28 (glutamate-dependent strain) under respective optimal fermentation conditions, the γ-PGA yield of GXG-5 was 19.0% higher than that of GXA-28, and GXG-5 was also superior to GXA-28 in the availability of carbon sources and substrates. Furthermore, the glutamate dependent difference between GXA-28 and GXG-5 was analyzed by genomic sequencing, results indicated that genes related to the glutamate dependent difference mainly involved in carbohydrate transport and metabolism and amino acid metabolism, and 13 genes related to γ-PGA synthesis were mutated in GXG-5. This study provided a potential glutamate-independent strain to replace glutamate-dependent strain for γ-PGA production, and shared novel information for understanding the glutamate dependent difference at the genomic level.

**Electronic supplementary material:**

The online version of this article (10.1186/s13568-017-0512-0) contains supplementary material, which is available to authorized users.

## Introduction

Poly-γ-glutamic acid (γ-PGA) is a biopolymer that synthesized inside the microbial cell via amide linkages between the α-amino and γ-carboxylic groups of glutamic acid. It has wide applications in industry, agriculture, medicine, food, cosmetic and water treatment, based on its excellent biological properties such as non-toxicity, edibility, biocompatibility and biodegradability (Shih and Van 2001). Industrialized production of γ-PGA is the prerequisite for its large-scale applications. Thus, many studies have attempted to improve γ-PGA production, such as screening producer with high yield, high productivity and high conversion efficiency, selecting cheaper substrates, and optimizing the fermentation process (Ogunleye et al. 2015). Among them, it is vitally important to develop a excellent strain.

*Bacillus* species were the main γ-PGA producing bacteria. According to the different sources of substrate, some strains were designated as glutamate-dependent type because of them produce γ-PGA only in the medium containing exogenous l-glutamate, such as *B. subtilis* IFO 3335 (Goto and Kunioka 1992), *B. subtilis* F-2-01 (Kubota et al. 1993), *B. licheniformis* ATCC 9945a (Birrer et al. 1994), *B. subtilis* NX-2 (Xu et al. 2005), *B. licheniformis* WX-02 (Wei et al. 2010), and *B. subtilis* GXA-28 (Zeng et al. 2014). While others designated as glutamate-independent type which could de novo synthesize γ-PGA from glucose, such as *B. licheniformis* A35 (Cheng et al. 1989), *B. subtilis* TAM-4 (Ito et al. 1996), *B. amyloliquefaciens* LL3 (Cao et al. 2011), and *B. subtilis* C10 (Zhang et al. 2012). Studies about γ-PGA production in the past few decades were mainly focused on glutamate-dependent strains. However, the high production cost caused from adding large quantities of exogenous l-glutamate was still the major obstacle to limit the large-scale production of γ-PGA. Therefore, many researchers had begun to focus on glutamate-independent strains at present, as these strains could significantly reduce the cost of γ-PGA production and simplify the fermentation process. While there were some defects such as low conversion rate of substrate in the currently reported glutamate-independent strains, we continue to believe that screening glutamate-independent strains in *Bacillus* with excellent fermentation characteristics was still a feasible method to improve γ-PGA production.

Current studies suggested that the synthesis process of γ-PGA in bacteria comprises three stages at least, including selection and transport of substrate, synthesis of γ-PGA, transport and degradation of γ-PGA (Ogunleye et al. 2015). Thereinto, the selection of substrate types (glucose or l-glutamate) directly affects the cost of γ-PGA production. However, there are few studies on the difference of substrate between glutamate-dependent strain and glutamate-independent strain. Furthermore, the molecular mechanism of this glutamate dependent difference also remains unclear. If contrastive studies are conducted between glutamate-dependent strain and glutamate-independent strain from fermentation level, cell physiology level and genome level, it is possible to provide some information for understanding this glutamate dependent difference.

According to above ideas, a novel glutamate-independent strain *B. subtilis* GXG-5 with thermotolerant characteristics was obtained, and its product was also characterized. Then, effects of carbon sources, nitrogen sources, temperature and pH on γ-PGA production were optimized, and scaled-up produced in 10 L fermentor. Furthermore, the difference of substrate between glutamate-dependent strain (*B. subtilis* GXA-28) and glutamate-independent strain (*B. subtilis* GXG-5) was analyzed based on the γ-PGA fermentation parameters and genomic sequencing. To our knowledge, it is the first report that efficient production of γ-PGA by a thermotolerant glutamate-independent strain and comparative genome analysis of the glutamate dependent difference.

## Materials and methods

### Microorganism and medium

Strain GXG-5 was a glutamate-independent γ-PGA producing strain, which isolated from soil samples of the farm in Guangxi University (Guangxi, China), and deposited at China Center for Type Culture Collection with an Accession Number of CCTCC M 2017083. The seed medium contained glucose 10 g/L, yeast extract 5 g/L, tryptone 10 g/L, and NaCl 10 g/L. The basal fermentation medium composed of glucose 25.0 g/L, soy peptone 3.0 g/L, NH_4_NO_3_ 17.0 g/L, NaCl 5.0 g/L, K_2_HPO_4_ 2.5 g/L, and MgSO_4_·7H_2_O 1.0 g/L. Initial pH was adjusted to 7.0 ± 0.1.

*Bacillus subtilis* GXA-28 (CCTCC M 2012347) was used as glutamate-dependent γ-PGA producer (Zeng et al. 2013), which produce γ-PGA with fermentation medium composed of glucose 30.0 g/L, yeast extract 2.5 g/L, l-glutamate 20.0 g/L, KH_2_PO_4_ 0.5 g/L, and MgSO_4_·7H_2_O 0.1 g/L. Initial pH was adjusted to 7.2 ± 0.1.

### Identification of the strain

Strain GXG-5 was characterized and identified by morphological, physiological, biochemical tests and 16S rDNA sequence method. The morphological characteristic was observed through an Olympus E330 camera (Olympus Corp., Japan) and a Hitachi SU-8020 scanning electron microscope (SEM; Hitachi Science Systems Ltd., Japan). Physiological and biochemical identification was performed according to Bergey’s Manual of Systematic Bacteriology (Claus and Berkeley 1986). The 16S rDNA gene was amplified by polymerase chain reaction (PCR) using universal primers, 1540r (5′-AGGAGGTGATCCAGCCGCA-3′) and 7f (5′-CAGAGTTTGAT CCTGGCT-3′). After sequencing, the 16S rDNA sequence was aligned with published sequences from the NCBI database using Clustal X program (http://www.clustal.org). The phylogenetic tree was constructed by the neighbor-joining method with MEGA 7 software based on the tests with 1000 bootstrap replicates (Kumar et al. 2016). Subsequently, the sequence was deposited at GenBank.

### Purification and characterization of the polymer

The polymer was recovered and purified according to the method reported previously (Goto and Kunioka 1992). The purified polymer and a γ-PGA standard were dissolved in deionized water (1 mg/mL), and scanned from 190 to 390 nm using a Multiskan GO UV/Vis microplate spectrophotometer (Thermo Scientific, USA) with deionized water as a baseline. The purified polymer was hydrolyzed by 6 M HCl at 105 °C for 8 h in a sealed and evacuated tube, neutralized with 6 M NaOH and then analyzed by thin layer chromatography (TLC) on Silica Gel-60 plate (Merck, Germany) using *n*-butanol-acetic acid-pyridine-water (4:1:1:2) as developing solvent and glutamate as a standard. The plate was dried and sprayed with acetone containing 0.2% ninhydrin to visualize the amino acid (Kambourova et al. 2001). The purified polymer dissolved in D_2_O solution was analyzed with a Nuclear Magnetic Resonance Spectrometer (AVANCE 600, Bruker Corp., Switzerland) at 600 MHz, and the ^1^H-NMR spectroscopy of the purified polymer was compared with that of a standard γ-PGA.

### Optimization of γ-PGA production

#### Effects of selected medium components on γ-PGA production

In order to investigate the effects of carbon and nitrogen sources on the γ-PGA production, nine carbon sources (glucose, fructose, sucrose, maltose, lactose, glycerol, mannitol, citric acid, and soluble starch) with concentration of 25.0 g/L, ten organic nitrogen sources (peptone, tryptone, soy peptone, yeast extract, beef extract, corn pulp powder, casein, wheat bran extract, soybean meal, and urea) with concentration of 3.0 g/L, and three inorganic nitrogen sources (ammonium sulfate, ammonium chloride, and ammonium nitrate) with concentration of 17.0 g/L were screened by single-factor test. Moreover, effects of glucose and ammonium nitrate concentrations on the γ-PGA production were also investigated in detail. The optimization experiments were carried out in shake flask at 37 °C and 200 rpm.

#### Effects of pH and temperatures on γ-PGA production

A range of pH (from 5.0 to 9.0) and temperatures (from 30 to 50 °C) were investigated to evaluate their effects on γ-PGA production, based on above optimized medium. Other conditions were consistent with the experiment of medium optimization.

### Cultivation condition in a 10 L fermentor

The GXG-5 cells were inoculated into 100 mL of seed medium in 500 mL flask and aerobically cultured at 50 °C for 12 h with shaking at 200 rpm. The seed culture (200 mL) was inoculated into 7 L initial fermentation medium in a 10 L fermentor (BLBIO, China) to start the cultivation at 50 °C. The pH was automatically controlled at 7.0 ± 0.1 by adding 2 N NaOH and/or 2 N HCl. The aeration rate was maintained at 0.5 vvm and the agitation speed was gradually increased from 200 to 300 rpm to increase dissolved oxygen. Besides, 150 μL antifoam (Sigma, USA) was added at the beginning of fermentation to control the formation of foam.

### Comparative genome analysis of the glutamate dependent difference between GXA-28 and GXG-5

#### Genomic DNA extraction

Genomic DNA of strain GXA-28 and GXG-5 were extracted from Luria Broth culture using the Ezup Column Bacteria Genomic DNA Purification Kit (Sangon, B518255), then the purity were detected by Nanodrop 2000 and agarose gel electrophoresis.

#### Sequencing

The sequencing process was carried out according to the standard protocol of Illumina, including sample quality testing, library construction, library quality testing and library sequencing. Briefly, genomic DNA was fragmented to an average length of 200–500 bp by the Covaris S220 system (Covaris, Woburn, MA) after qualified. The fragmented DNA was purified and repaired, a single A nucleotide was ligated to the 3′ end, Illumina Index PE adapters (Illumina, San Diego, CA) were ligated to the fragments, and the sample was size-selected aiming for a 300-bp product with E-Gel SizeSelect Agarose Gels 2% (Invitrogen, Grand Island, NY). The size-selected product was amplified by PCR for 18 cycles with primers InPE1.0, InPE2.0, and Index primer containing a unique-index tag for each individual sample. The final product was validated by Agilent Bioanalyzer 2100 (Agilent, Santa Clara, CA). Pooled libraries were sequenced on Illumina Hiseq X-Ten.

#### Analysis of sequences

The clean reads were obtained from raw reads by removing adapter reads, “N”-rich reads and duplicate reads, then mapping with the reference genome sequence of *B. subtilis* 168 (Accession Number: NC 000964.3) by Burrows–Wheeler aligner (BWA) software (Li and Durbin 2009). Single nucleotide polymorphism (SNP) and Insertion deletion (InDel) were detected by GATK software toolkit (McKenna et al. 2010), and Structure variation (SV) was analyzed by BreakDancer software (Chen et al. 2009). Based on the non-synonymous SNP, InDel and SV in coding sequence region, the genes with possible functional difference between GXA-28 and GXG-5 were presented. GO (Gene Ontology), COG (Clusters of Orthologous Groups of proteins) and KEGG (Kyoto Encyclopedia of Genes and Genomes) annotations for these variants were performed for gene and protein function prediction (Ashburner et al. 2000; Kanehisa et al. 2004; Tatusov et al. 2000). In addition, genes related with γ-PGA synthesis were also analyzed.

#### Nucleotide sequence accession number

Raw sequence data of *B. subtilis* GXA-28 and GXG-5 have been deposited in the NCBI Short Read Archive with Accession Numbers SRX3083478 and SRX3083516, respectively.

### Analytical methods

The cell growth was determined by optical density (OD) at 660 nm (Cao et al. 2011). The γ-PGA yield was measured by UV or HPLC method (Zeng et al. 2012). The concentration of glucose was measured by a biosensor equipped with glucose oxidase electrode (SBA-40D, Shandong Academy of Sciences, China). Each experiment was carried out in triplicates, and three parallel experiments were performed for each experiment.

## Results

### Isolation and identification of the strain GXG-5

**Fig. 1:**
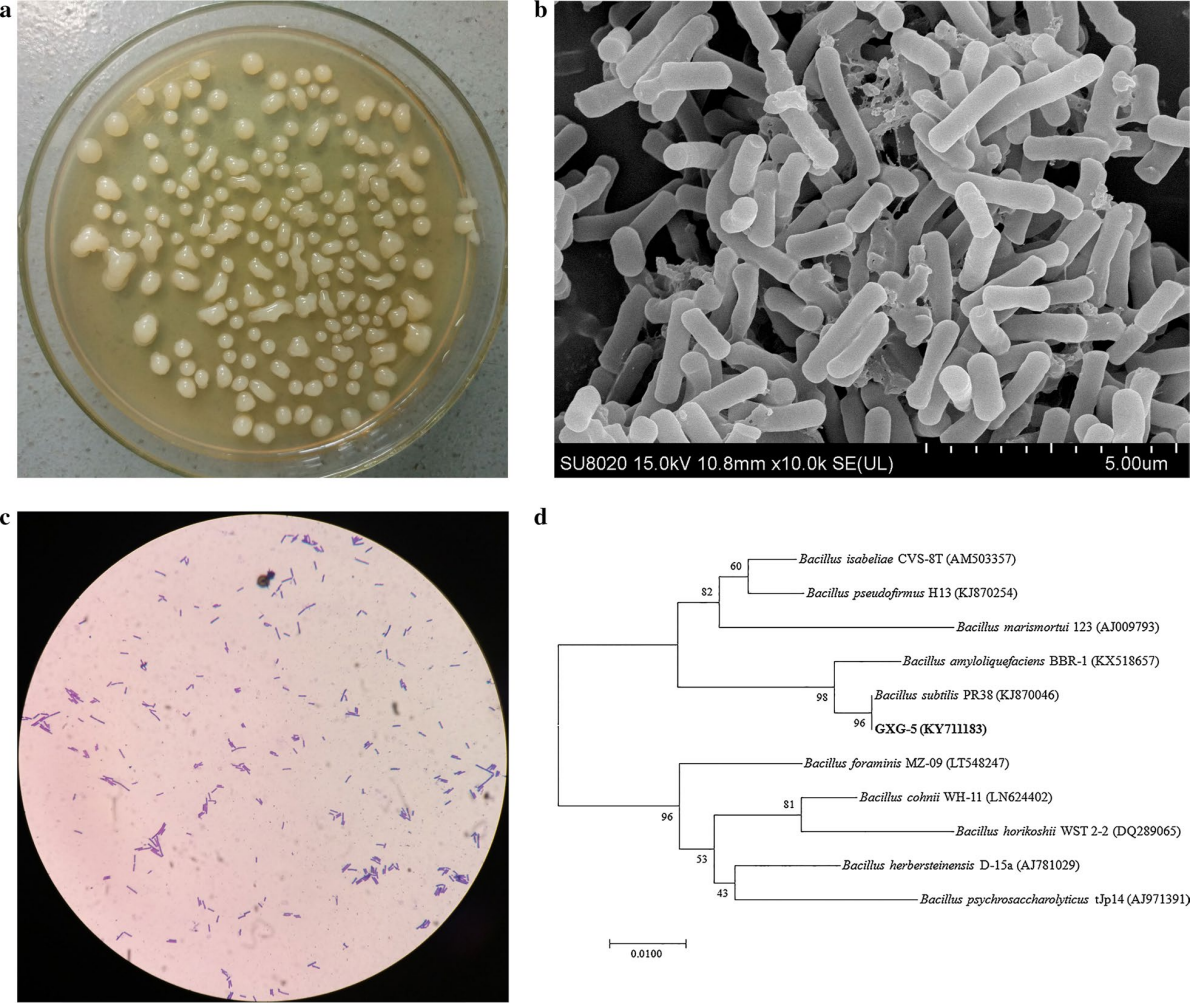
**a** The colonial morphology of strain GXG-5 on agar plate. The plate medium composed of glucose 25.0 g/L, soy peptone 3.0 g/L, NH_4_NO_3_ 17.0 g/L, NaCl 5.0 g/L, K_2_HPO_4_ 2.5 g/L, and MgSO_4_·7H_2_O 1.0 g/L. Initial pH was adjusted to 7.0 ± 0.1. **b** The microscopic features of GXG-5 (scanning electromicroscope, ×10,000). **c** Gram staining of GXG-5 (optical microscope, Olympus CX41, ×100). **d** Neighbor-joining phylogenetic tree based on 16S rDNA gene sequences showing the position of strain GXG-5 (GenBank ID: KY711183) among its closely related organisms. Numbers in parentheses are NCBI accession numbers of published sequences. Bootstrap values (1000 replicates) are mentioned at the nodes. The scale bar represents 0.01 nucleotide substitution per position

About 300 highly mucoid colonies were picked up from the screening plate, and then transferred into the basal fermentation medium. The strain GXG-5 was able to produce highest viscous culture broth, and it was selected for the following study. The 1447 bp fragment of 16S rRNA gene was amplified, sequenced and submitted to the GeneBank (Accession Number KY711183). Comparison of the obtained sequence with other sequences available at NCBI database revealed 100% identity to the corresponding sequence of *B. subtilis* PR38 (KJ870046), *B. subtilis* SQL 01 (KF051998), and *B. subtilis* XJG2-1 (JX502843). A phylogenetic tree was constructed based on neighbor-joining method using MEGA 7 software, and showed that the strain is more related to *B. subtilis* (Fig. 1d).

The colonial morphology of GXG-5 was circular, moist and looked like a water-drop with smooth surface and regular edge (Fig. 1a). The SEM mage of GXG-5 cells showed rod-shape with size of 0.5–0.7 × 1.0–3.0 μm (Fig. 1b). Gram staining showed that GXG-5 was a Gram-positive bacterium (Fig. 1c). The physiological and biochemical characteristics of GXG-5 were summarized in Additional file 1: Table S1. It showed that GXG-5 could assimilate a variety of carbohydrates and hydrolyze starch, casein and gelatin. GXG-5 could also utilize urea and ammonium salts as nitrogen source. Furthermore, GXG-5 could grow in a wide range of pH (from 5.0 to 9.0) and on the LB solid medium contained 10% NaCl (w/v) at 50 °C. Based on above morphological, physiological, biochemical tests and 16S rRNA sequence analysis, the strain GXG-5 was identified as *B. subtilis* with thermotolerant characteristics, and deposited at China Center for Type Culture Collection with an Accession Number of CCTCC M 2017083.

### Characterization of the polymer

Result of TLC showed that the hydrolysate of purified polymer with a single spot identical to that of authentic glutamate, and there are no free glutamates in the purified polymer (Additional file 1: Figure S1). The UV scanning spectrum of purified polymer presented the absorption peak at 216 nm is the same as that of the standard γ-PGA sodium salt. It does not exhibit absorption peak in the range of 260–280 nm, indicating that the polymer does not have a typical peptide chain structure. Furthermore, the ninhydrin and biuret reactions for the purified polymer were negative. The phenol–sulfuric acid method showed that no polysaccharide was detected in the purified polymer. The ^1^H-NMR spectrum of the purified polymer was consistent with that of the standard γ-PGA sodium salt (Additional file 1: Figure S2). In addition, the purified polymer is very soluble in water or aqueous solution at neutral pH conditions, while γ-PGA (H form) is not soluble in water but only the organic solvent-DMSO (Ho et al. 2006). All of above results suggested that γ-PGA was the product of *B. subtilis* GXG-5, and the γ-PGA belongs to Na^+^ form.

### Optimization of γ-PGA production by *B. subtilis* GXG-5

**Fig. 2:**
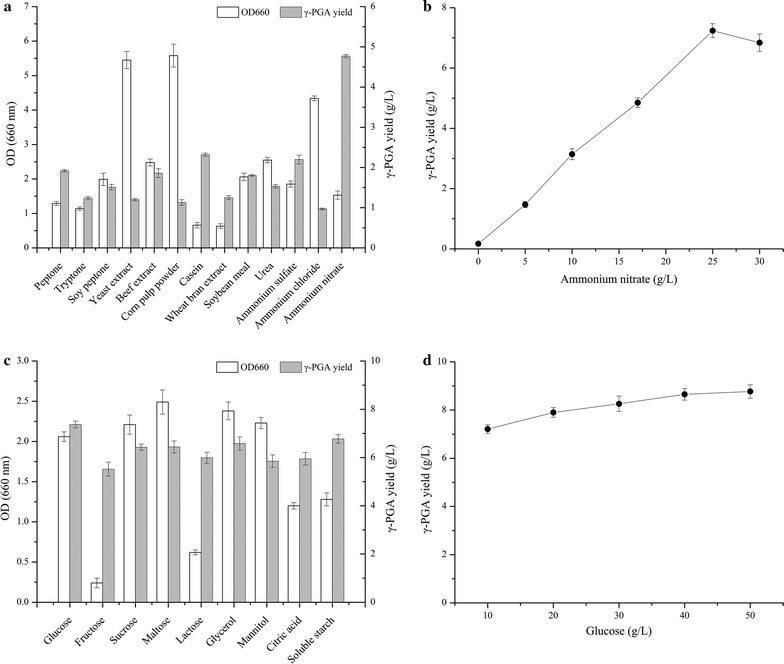
Effects of **a** different nitrogen sources, **b** different ammonium nitrate concentrations, **c** different carbon sources, and **d** different glucose concentrations on cell growth and γ-PGA yield in *B. subtilis* GXG-5

Owing to the organic nitrogen source and inorganic nitrogen source were added simultaneously in the basal fermentation medium, the effects of nitrogen sources on γ-PGA production were investigated firstly. As shown in Fig. 2a, *B. subtilis* GXG-5 was able to produce γ-PGA from a single nitrogen source, and showed widespread availability of nitrogen sources. Among which, yeast extract and corn syrup powder were more beneficial to cell growth than other nitrogen sources. Interestingly, casein was not conducive to the cell growth, but it could promote cell to synthesize the highest yield of γ-PGA in organic nitrogen sources. This indicated that there was no positive correlation between cell growth and product synthesis. The maximum yield of γ-PGA was 4.77 ± 0.14 g/L, which was obtained from medium supplemented with inorganic nitrogen of ammonium nitrate. Furthermore, effects of ammonium nitrate concentration on γ-PGA production was studied (Fig. 2b). The γ-PGA yield was increased with the ammonium nitrate concentration range of 0–25 g/L, and the maximum yield of γ-PGA reached 7.24 ± 0.23 g/L. When the addition of ammonium nitrate exceeded 25 g/L, the γ-PGA yield decreased slightly. Based on above results, 25 g/L ammonium nitrate was selected as the only nitrogen source for further study.

In order to investigate the effect of exogenous l-glutamate on the γ-PGA production by *B. subtilis* GXG-5, l-glutamate as a carbon source was added to the fermentation medium, but GXG-5 was not observed to produce γ-PGA. In contrast, as the l-glutamate was replaced by other carbon sources, such as glucose, fructose, sucrose, maltose, lactose, glycerol, mannitol, citric acid and soluble starch, a large amount of γ-PGA were detected in the fermentation broth. Thus, *B. subtilis* GXG-5 could be identified as a glutamate-independent γ-PGA producing strain. As shown in Fig. 2c, although there were some differences in cell growth with various carbon sources, the γ-PGA yield varied slightly between 5.5 and 7.4 g/L. The maximum yield of γ-PGA was 7.37 ± 0.16 g/L when glucose used as carbon sources. In addition, there was no obvious change in γ-PGA yield when the glucose concentration was between 10 and 50 g/L. Thus, 25 g/L glucose was chose as the carbon source for further study.

**Fig. 3:**
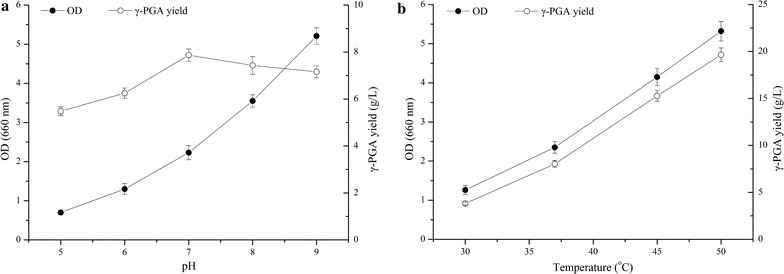
Effects of **a** different pH, and **b** different temperatures on cell growth and γ-PGA yield in *B. subtilis* GXG-5

Effects of pH and temperatures on γ-PGA production were also studied (Fig. 3). The cell growth was promoted by pH from 5.0 to 9.0, but the maximum yield of γ-PGA was obtained at pH 7.0. It is interesting that the cell growth and γ-PGA yield were improved with temperature from 30 to 50 °C. The maximum cell growth and γ-PGA yield were reached 5.32 ± 0.25 (OD_660nm_) and 19.65 ± 0.73 g/L at 50 °C, respectively. Thus, pH 7.0 and 50 °C were selected for further study.

### Scaled-up production of γ-PGA by *B. subtilis* GXG-5 in 10 L fermentor

**Fig. 4:**
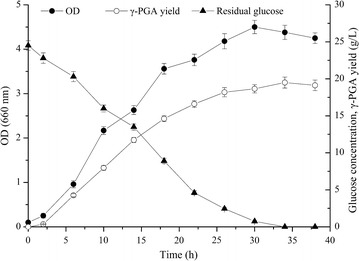
Time courses of batch γ-PGA production in 10 L fermentor by *B. subtilis* GXG-5

In order to further validate the above optimized medium components and fermentation conditions, the production of γ-PGA was carried out in a 10 L fermentor. As shown in Fig. 4, the time course of several important parameters including cell growth, γ-PGA yield and residual glucose were monitored during the batch fermentation. The γ-PGA yield reached 19.50 ± 0.75 g/L with productivity of 0.57 g/L/h at 34 h, which comparable to that of flask fermentation. The cell growth increased rapidly during the first 18 h and the maximum value reached 4.38 ± 0.16 (OD_660nm_) at 30 h. It showed that the patterns of γ-PGA synthesis was essentially consistent with cell growth. Glucose had maintained high consumption rate during the whole fermentation process, because it was not only the material basis of cell growth, but also the substrate source of γ-PGA synthesis. After 34 h, the glucose was depleted and the cell growth was also decreased slightly.

### Whole-genome sequencing

In order to investigated the glutamate dependent difference between glutamate-dependent strain and glutamate-independent strain from the genome-wide level, the genome of GXA-28 and GXG-5 were sequenced. A total of 1 Gb sequencing data of GXA-28 as well as GXG-5 were generated by Illumina Hiseq X-Ten platform. As shown in Additional file 1: Table S2, 3,919,388 and 4,727,445 clean reads were obtained after filtering, then 96.01 and 95.15% of reads from GXA-28 and GXG-5 were mapped to the reference sequence of *B. subtilis* 168, respectively. In these two samples, the sequencing depth of genome was more than 280× and the coverage of genome was above 92%. The genome data was qualified for the further analysis with the Q20 > 95.49% and Q30 > 89.37%.

### Identification and annotation of SNPs, InDels and SVs

Compared with the reference genome, 23,534 SNPs, 916 InDels and 167 SVs were identified in GXA-28, while 30,031 SNPs, 1084 InDels and 187 SVs were detected in GXG-5 by GATK software toolkit (Additional file 1: Tables S3–S5). Thereinto, 12,229 SNPs and 476 InDels were identified both in GXA-28 and GXG-5. It is significant that 17,802 SNPs, 608 InDels and 187 SVs were unique to GXG-5 (Additional file 1: Figure S3).

**Fig. 5:**
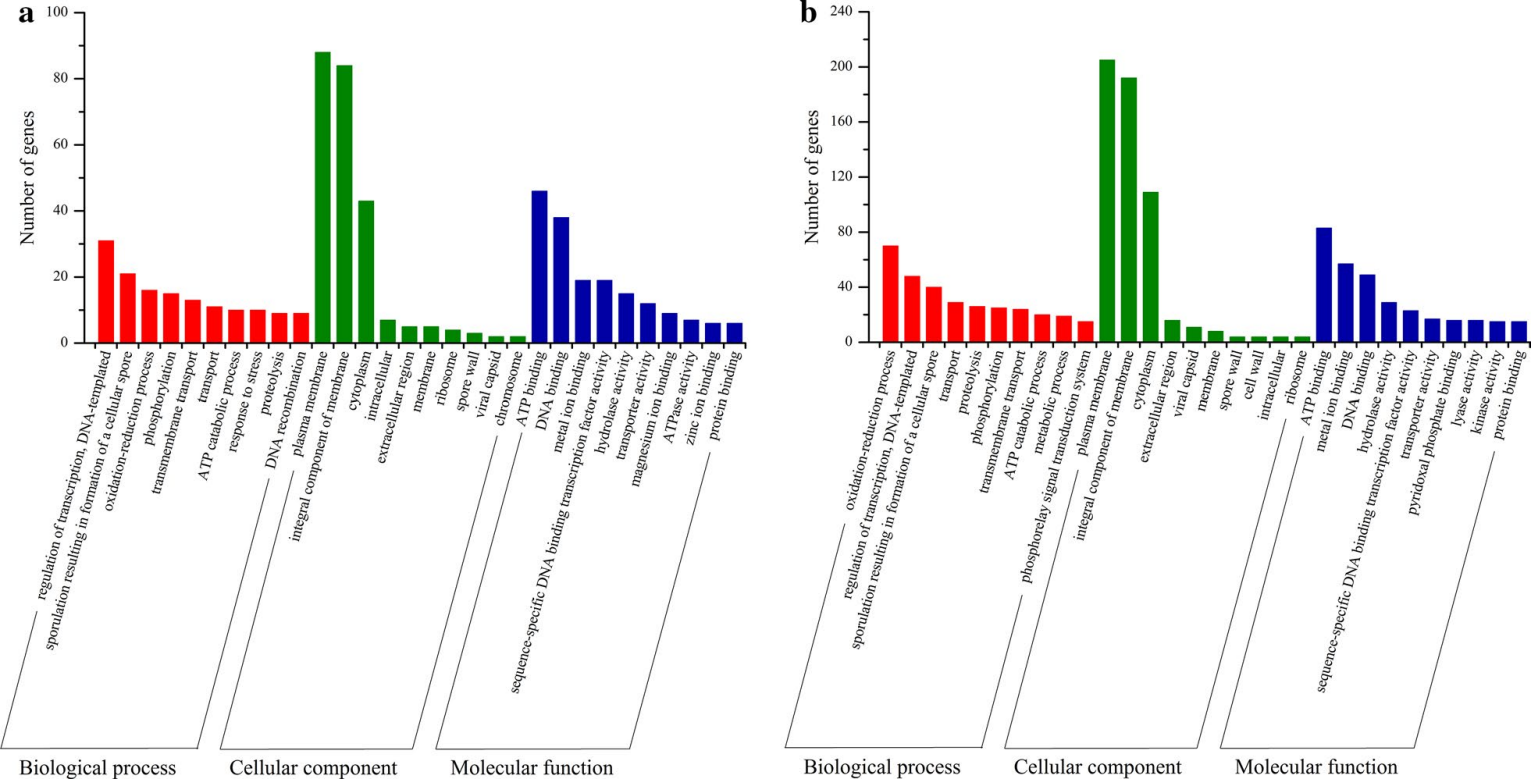
GO classification of the genes with possible functional difference between GXA-28 and GXG-5. **a** GXA-28; **b** GXG-5

**Fig. 6:**
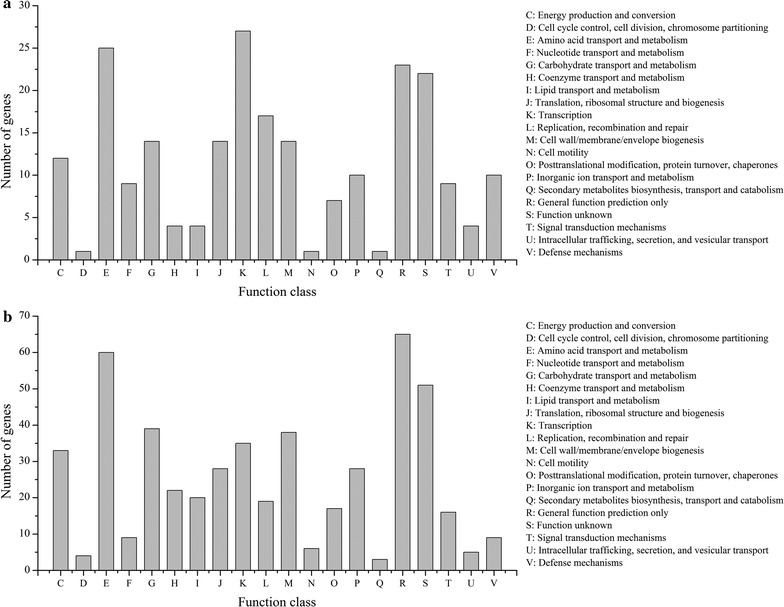
COG annotation of the genes with possible functional difference between GXA-28 and GXG-5. **a** GXA-28; **b** GXG-5

**Fig. 7:**
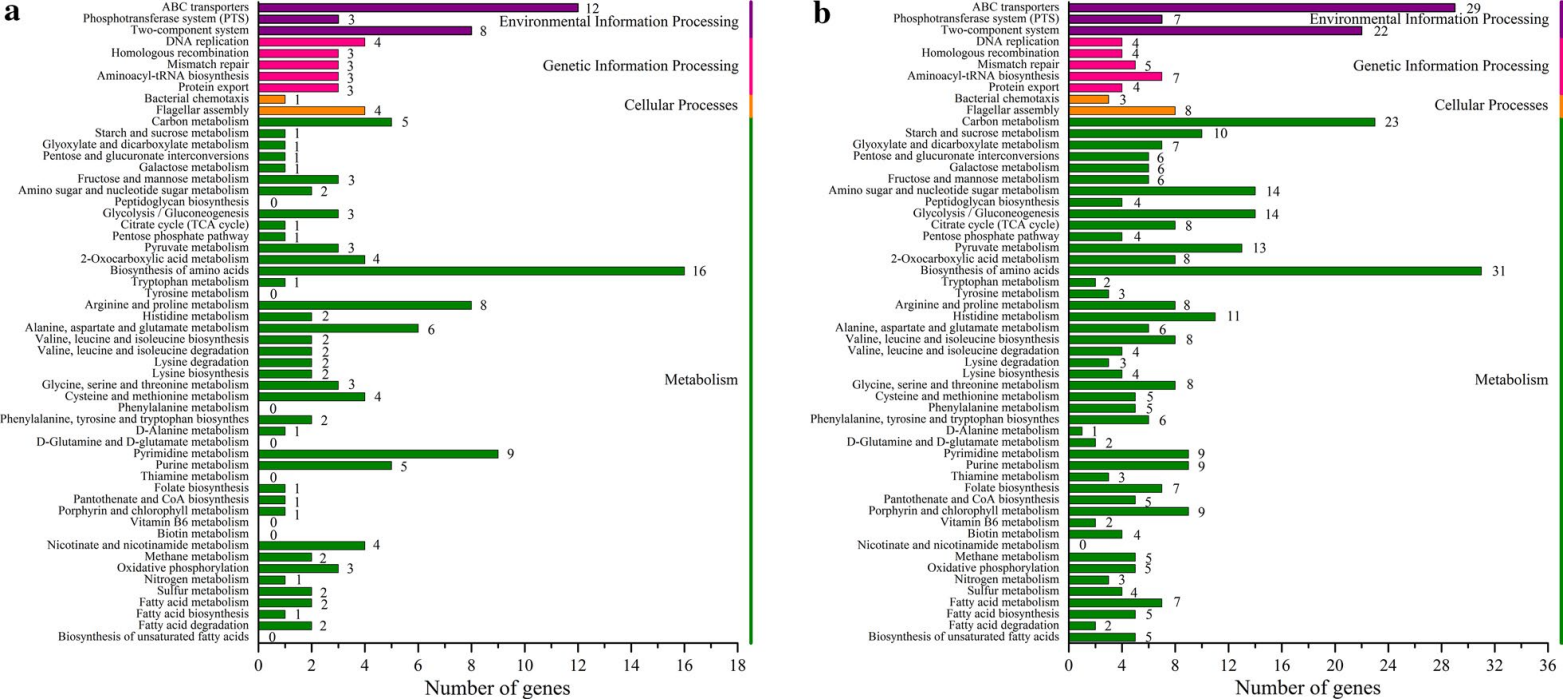
KEGG annotation of the genes with possible functional difference between GXA-28 and GXG-5. **a** GXA-28; **b** GXG-5

Based on above SNPs, InDels and SVs, 1743 and 2157 variant genes were obtained from GXA-28 and GXG-5 after comparing to the reference genome, respectively (Additional file 1: Table S6). Among them, 1383 genes were mutated in both strains, while 774 genes were mutated only in GXG-5, which much more than that of 360 genes in GXA-28 (Additional file 1: Figure S3). Subsequently, all differential mutated genes of GXA-28 and GXG-5 were annotated by GO, COG and KEEG. GO is an international standardized classification system of gene functions. As shown in Fig. 5, ten subcategories per GO category were listed. In biological process category, 70 mutant genes of GXG-5 were classified to the oxidation–reduction process, while only 16 mutant genes in GXA-28. Regarding cellular component category, the number of mutant genes in the top three subcategories were much more in GXG-5. Interestingly, similar phenomena were also present in the molecular function category. The COG database is designed to find homologous genes. 228 genes of GXA-28 and 507 genes of GXG-5 were annotated and classified into 20 subcategories (Fig. 6). Except for the subcategories of function unknown (S), transcription (K), amino acid transport and metabolism (E), and general function prediction only (R) were the largest group in GXA-28, while general function prediction only (R), amino acid transport and metabolism (E), and carbohydrate transport and metabolism (G) were the top three subcategories in GXG-5. The KEGG is a database for analyzing the metabolic pathways of gene products in cells and the functions of these gene products. In GXG-5, biosynthesis of amino acids, ABC transporters, and carbon metabolism were the largest subcategories involved 31, 29 and 23 unigenes, respectively, which more than that of in GXA-28 (Fig. 7).

### Analysis of genes related to the γ-PGA synthesis

In order to further investigate the glutamate dependent differences between GXA-28 and GXG-5, genes related to the γ-PGA synthesis were extensively analyzed according to the previous literature (Ashiuchi et al. 1999; Feng et al. 2015; Kimura et al. 2004; Stanley and Lazazzera 2005; Tran et al. 2000; Wu et al. 2011; Yamashiro et al. 2011). As shown in Additional file 1: Figure S4, these genes were involved in specific processes include glucose transport (*glc*T, *pts*G, *pts*H, *pts*I, *glc*P, *glc*U), ammonium transport (*nrg*A, *nrg*B), exogenous l-glutamate transport (*glt*T, *glt*P), α-oxoglutarate node of TCA cycle (*icd*, *odh*A), glutamate synthesis (*gln*A, *glt*B, *glt*A, *gud*B, *roc*G, *yrp*C, *rac*E), γ-PGA synthesis (*pgs*B, *pgs*C, *pgs*A, *pgs*E), regulation of γ-PGA synthesis (*com*Q, *com*X, *com*P, *com*A, *deg*S, *deg*U, *deg*Q, *swr*AA). Compared with GXA-28, 13 genes were mutated in GXG-5 (Additional file 1: Table S7). With the exception of *pts*H, other genes were mutated in varying degrees in the process of glucose transport, especially in *glc*P which had four non-synonymous mutations. Single nucleotide change was found in AmtB (encoded by *nrg*A), which was responsible for the ammonium transport at low ammonium concentrations and at low pH (Gunka and Commichau 2012). For glutamate-independent γ-PGA producing bacteria, the process of endogenous glutamate synthesis was critical. There were six genes related to glutamate synthesis mutated in GXG-5. Among which, six nucleotide changes were found in *glt*A, and three codon deletions were presented in *gud*B. In addition, two SVs of insertion and deletion occurred in *glt*B and *yrp*C, respectively. These changes of genes related to glucose transport, ammonium transport and glutamate synthesis may be the cause of the glutamate dependent difference. Interestingly, no nucleotide changes were found in genes related to exogenous l-glutamate transport, γ-PGA synthesis and regulation of γ-PGA synthesis, except for *pgs*E. These results indicated that there was no obvious difference in the ability of γ-PGA synthesis between GXA-28 and GXG-5. This was also illustrated by the γ-PGA yield of GXA-28 and GXG-5 in 10 L fermentor (Table 2).

## Discussion

Comparing the γ-PGA production between GXG-5 with other glutamate-independent strains (Table 1). Although the γ-PGA yield was not the highest, the strain GXG-5 could convert the most of carbon source to γ-PGA in a relatively short time (34 h). The conversion efficiency of carbon source was 78% in GXG-5, indicating that GXG-5 could save more fermentation raw material cost than other strains. As shown in Table 2, only the carbon source was considered, the cost were calculated for production of 10 kg γ-PGA by different glutamate-independent strains. The cost was estimated to be $7.49 in *B. subtilis* GXG-5, which was reduced by 62.21–93.75% as compared with other glutamate-independent strains. In the reported literature, the optimal fermentation temperature of γ-PGA production was generally between 30 and 40 °C (Cao et al. 2011; Wei et al. 2010; Xu et al. 2005; Zhang et al. 2012). Significantly, GXG-5 could produce γ-PGA under high temperature of 50 °C. As we all know, high temperature fermentation not only decreases the sterilization cost, but also reduces the contamination possibilities. Compared with the conventional fermentation, the consumption of cooling water can also greatly reduce in high temperature fermentation, which leads to lower energy consumption and production cost for large-scale industrial fermentation, especially in tropical or subtropical areas. In addition, operating a fermentor at elevated temperatures may be advantageous in terms of increased solubility of substrates, improved mass transfer due to decreased viscosity, and increased diffusion rates. Thus, such a high conversion rate of substrate and fermentation temperature provided support for industrial production of γ-PGA by glutamate-independent strain in the future.

**Table 1:** Comparison of γ-PGA production in *Bacillus* with glutamate-independent

Strains	Medium components		Fermentation conditions			Yield (g/L)	Conversion efficiency (%)^a^	References
	Carbon source	Nitrogen source	Bioreactor	Temperature (°C)	Time (h)			
*B. amyloliquefaciens* LL3	Sucrose (50 g/L)	(NH_4_)_2_SO_4_ (2 g/L)	200 L	37	44	4.4	8.80	Cao et al. (2011)
*B. licheniformis* A35	Glucose (75 g/L)	NH_4_C1 (18 g/L)	Flask	30	120	12.0	16.00	Cheng et al. (1989)
*B. subtilis* TAM-4	Glucose (75 g/L)	NH_4_C1 (18 g/L)	Flask	30	96	22.1	29.47	Ito et al. (1996)
*B. subtilis* C10	Glucose (80 g/L) Citric acid (20 g/L)	NH_4_C1 (10 g/L)	10 L	32	32	27.7	27.70	Zhang et al. (2012)
*B. subtilis* GXG-5	Glucose (25 g/L)	NH_4_NO_3_ (25 g/L)	10 L	50	34	19.5	78.00	This study

^a^The conversion efficiency was defined as the ratio of γ-PGA yield and carbon source concentration

**Table 2:** Comparison of the carbon source cost in different glutamate-independent strains to produce 10 kg γ-PGA

Strains	Carbon source				Cost saving (%)^a^
	Types	Price ($ t^−1^)	Amount (kg)	Cost ($)	
*B. amyloliquefaciens* LL3	Sucrose	1054	113.64	119.78	93.75
*B. licheniformis* A35	Glucose	584	62.50	36.50	79.48
*B. subtilis* TAM-4	Glucose	584	33.94	19.82	62.21
*B. subtilis* C10	Glucose	584	28.88	23.18	67.69
	Citric acid	875	7.22		
*B. subtilis* GXG-5	Glucose	584	12.82	7.49	–

^a^Represented the cost saving proportion of *B. subtilis* GXG-5 compared with the corresponding strain

*Bacillus subtilis* GXA-28 was a glutamate-dependent strain that isolated from marine sands by our lab (Zeng et al. 2013, 2014), while *B. subtilis* GXG-5 was a glutamate-independent strain. The two strains all have the ability to produce γ-PGA under high temperature condition (50 °C), but only the substrate sources were different. The production of γ-PGA by these two strains with respective fermentation medium at 50 °C in 10 L fermentor were also compared. As shown in Table 3, the fermentation time of GXG-5 was longer than that of GXA-28, but the γ-PGA yield of GXG-5 (19.50 ± 0.75 g/L) was 19.0% higher than that of GXA-28. The cell growth of this two strains were similar, and the maximum OD_660nm_ were 4.82 ± 0.18 and 4.50 ± 0.15, respectively. Glucose was completely consumed and glutamate was not detected in the medium of GXG-5, but there were still a small quantity of residual glucose and glutamate in medium of GXA-28 at the end of fermentation. These results suggested that the glutamate-independent strain of GXG-5 was superior to the glutamate-dependent strain of GXA-28 in the availability of carbon sources and substrates. In addition, the GXG-5 was also close to GXA-28 in terms of γ-PGA productivity, especially in the conversion efficiency of substrate, even though the fermentation time of GXG-5 was extended by 12 h. Based on above results, GXG-5 as a glutamate-independent strain has the potential to replace glutamate-dependent strain for γ-PGA production.

**Table 3:** Comparative analysis of γ-PGA production at 50 °C in 10 L fermentor between GXA-28 and GXG-5

Strain	Maximum OD_660nm_	Maximum γ-PGA yield (g/L)	Residual glucose (g/L)	Residual l-glutamate (g/L)	Time (h)	γ-PGA productivity (g/L/h)	Conversion efficiency (%)
GXA-28	4.82 ± 0.18	16.39 ± 0.57	2.75 ± 0.23	4.38 ± 0.26	22	0.75	81.95^a^
GXG-5	4.50 ± 0.15	19.50 ± 0.75	0	–^c^	34	0.57	78.00^b^

^a^The conversion efficiency was defined as the ratio of γ-PGA yield and exogenous l-glutamate concentration

^b^The conversion efficiency was defined as the ratio of γ-PGA yield and glucose concentration

^c^No detect

Compared to the reference genome *B. subtilis* 168, large number of variations including SNPs, InDels and SVs were detected in *B. subtilis* GXA-28 and *B. subtilis* GXG-5, which similar to Kamada’s reports that there were many variations between wild *B. subtilis* strains isolated from non-salted fermented soybean foods and *B. subtilis* 168 (Kamada et al. 2015). The total variants of GXG-5 was more than that of GXA-28 in 27.16%, which may be suggest that more genes were mutated in glutamate-independent strain than in glutamate-dependent strain. In these variants, the number of SNPs were much more than that of InDels and SVs. Besides, nearly 83% SNPs were located in coding sequence (CDS) regions, while over 77% InDels were found in upstream and (or) downstream regulatory regions of two strains. This indicated that SNPs was the major mutation type causing phenotypic differences between GXA-28 and GXG-5. Furthermore, those different variants unique to GXG-5 probably resulted in the glutamate dependent difference between GXA-28 and GXG-5. In glutamate-independent strain, the substrate glutamate for γ-PGA synthesis need to be transformed from glucose via glycolytic pathway, TCA cycle and glutamate synthesis pathway (Zhang et al. 2012). The genes involved in these metabolic pathways generally belong to carbohydrate transport and metabolism and amino acid metabolism. Interestingly, the number of mutated genes related to carbohydrate transport and metabolism and amino acid metabolism in GXG-5 were 2.54 and 2.52 times that of GXA-28 by COG and KEGG annotation, respectively. Besides, results of mutated genes annotation in GXG-5 showed that the subcategories of carbohydrate transport and metabolism and amino acid metabolism were the largest group. Those indicated that genes related to the glutamate dependent differences probably mainly involved in carbohydrate transport and metabolism and amino acid metabolism. Therefore, the next step in the study of the glutamate dependent difference can be focused on these mutated genes.

In *B. subtilis*, glucose transport was realized by three different systems: a glucose-specific PTS permease (IICBA^Glc^, encoded by *pts*G, *pts*H, *pts*I and *glc*T), a hexose/H^+^ symporter (GlcP), and a glucose uptake protein (GlcU) (Jahreis et al. 2008). Besides, glucose transport was the first step in the use of glucose by bacteria. Compared to GXA-28, the key genes of above three glucose transport system all mutated in GXG-5, indicating that the existence of differences in the glucose utilization between glutamate-dependent strain and glutamate-independent strain. There are two pathways for glutamate synthesis: the glutamine synthesis–glutamate synthase (GS-GOGAT, encoded by *gln*A, *glt*B and *glt*A) pathway and the glutamate dehydorgenase (GDH, encoded by *gud*B or *roc*G) pathway (Gunka and Commichau 2012). In the process of glutamate synthesis, these two pathways both require ammonium to participate. However, due to the GS-GOGAT has a relatively high affinity for ammonium than GDH, the GS-GOGAT pathway play a more important role in the glutamate synthesis (Schreier 1993). In GXG-5, a pronounced mutation occurred in the glutamate synthase gene, especially a structural variation (insertion of a 285 bp fragment) presented in *glt*B. This may be suggested that the GS-GOGAT pathway play a different role in glutamate-independent strain. Ashiuchi et al. found exceptionally high levels of glutamate racemase (GLR, encoded by *yrp*C and *rac*E) activity in a natural strain of *B. subtilis* that can produce γ-PGA in abundance (Ashiuchi et al. 1998). Then, Kimura et al. reported that *rac*E is essential for growth in rich medium but dispensable for growth in minimal medium, where *yrp*C executes the anaplerotic role of *rac*E (Kimura et al. 2004). Interestingly, the sequence of *yrp*C was deleted in GXG-5, indicating that only RacE was used for the d-glutamic acid synthesis in glutamate-independent strain. These differential genes related to γ-PGA synthesis provided candidate genes for further research on the glutamate dependent difference between glutamate-dependent strain and glutamate-independent strain.

In conclusion, we have succeeded in obtaining a novel thermotolerant glutamate-independent strain *B. subtilis* GXG-5, and it has the potential to replace glutamate-dependent strain for γ-PGA production. In addition, we analyzed the glutamate dependent difference between GXA-28 and GXG-5 from fermentation level and genome level, then provided some novel information for understanding the glutamate dependent difference.

## Additional file

**Additional file 1.** Additional tables and figures.

## Abbreviations

γ-PGA: poly-γ-glutamic acid
*B*. *subtilis*: *Bacillus subtilis*
PCR: polymerase chain reaction
TLC: thin layer chromatography
^1^H-NMR: nuclear magnetic resonance hydrogen spectrum
UV: ultraviolet spectrum
HPLC: high performance liquid chromatography
SEM: scanning electron microscope
OD: optical density
DMSO: dimethyl sulfoxide
IICBA^Glc^: glucose-specific PTS permease
GlcP: hexose/H^+^ symporter
GlcU: glucose uptake protein
GS-GOGAT: glutamine synthesis–glutamate synthase
GDH: glutamate dehydrogenase
GLR: glutamate racemase
SNP: single nucleotide polymorphism
InDel: insertion deletion
SV: structure variation
GO: gene ontology
COG: clusters of orthologous groups of proteins
KEGG: kyoto encyclopedia of genes and genomes
CDS: coding sequence
TCA: tricarboxylic acid
BWA: Burrows–Wheeler aligner
