# Supplementary material for: Production of poly-γ-glutamic acid by a thermotolerant glutamate-independent strain and comparative analysis of the glutamate dependent difference
Source: AMB Express. 2017 Nov 25;7:213. doi: 10.1186/s13568-017-0512-0 (PMC5701898; doi:10.1186/s13568-017-0512-0)
Supplement: Supplementary file 1 — Additional file 1. Additional tables and figures. [file 13568_2017_512_MOESM1_ESM.docx]

**Additional Material**

**Article title:** Production of poly-γ-glutamic acid by a thermotolerant glutamate- independent strain and comparative analysis of the glutamate dependent difference

# Journal name: AMB Express

**Authors:** Wei Zeng^1,2#^, Guiguang Chen^2#^, Ye Guo^1,2#^, Bin Zhang^1,2^, Mengna Dong^1,2^, Yange Wu^1,2^, Jun Wang^1,2^, Zhiqun Che^1,2^, Zhiqun Liang^1,2^*

^1^State Key Laboratory for Conservation and Utilization of Subtropical Agro-bioresources, Guangxi University, Nanning 530004, Guangxi, China

^2^College of Life Science and Technology, Guangxi University, Nanning 530004, Guangxi, China

***Correspondence author.**

Zhiqun Liang,

State Key Laboratory for Conservation and Utilization of Subtropical Agro-bioresources, College of Life Science and Technology,

Guangxi University, 100 Daxue Road, Nanning, Guangxi, China

Post code: 530004. Tel. / fax: +86 0771 3271181.

E-mail address: [zqliang@gxu.edu.cn](mailto:zqliang@gxu.edu.cn)

^#^Wei Zeng, Guiguang Chen and Ye Guo contributed equally to this work.

**Fig. S1.** Thin layer chromatography analysis of the purified polymer. Lane 1, L-glutamate solution; Lane 2, L-glutamate and hydrolyzed polymer mixed-solution; Lane 3, hydrolyzed polymer solution; Lane 4, polymer solution.

**
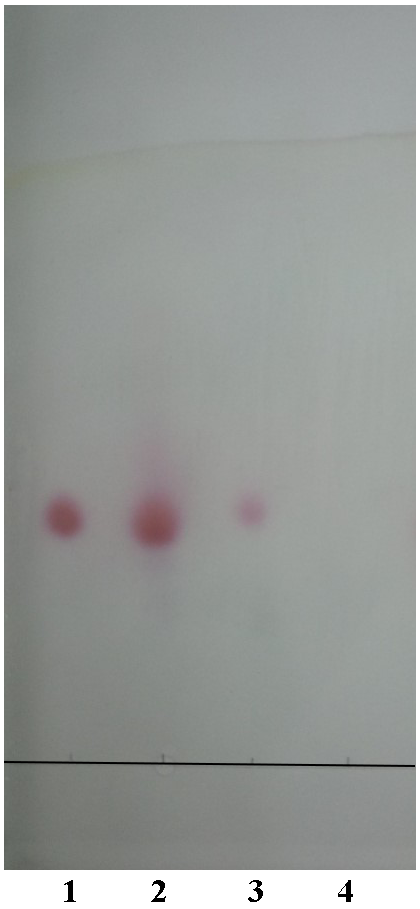
**

**Fig. S2.** 600 MHz ^1^H-NMR spectrum of the standard γ-PGA obtained from Wako Pure Chemical Industries (a) and the purified polymer (b) in D_2_O. The chemical shifts of α-H, β-H, γ-H and N-H were labeled in the peak station.

**
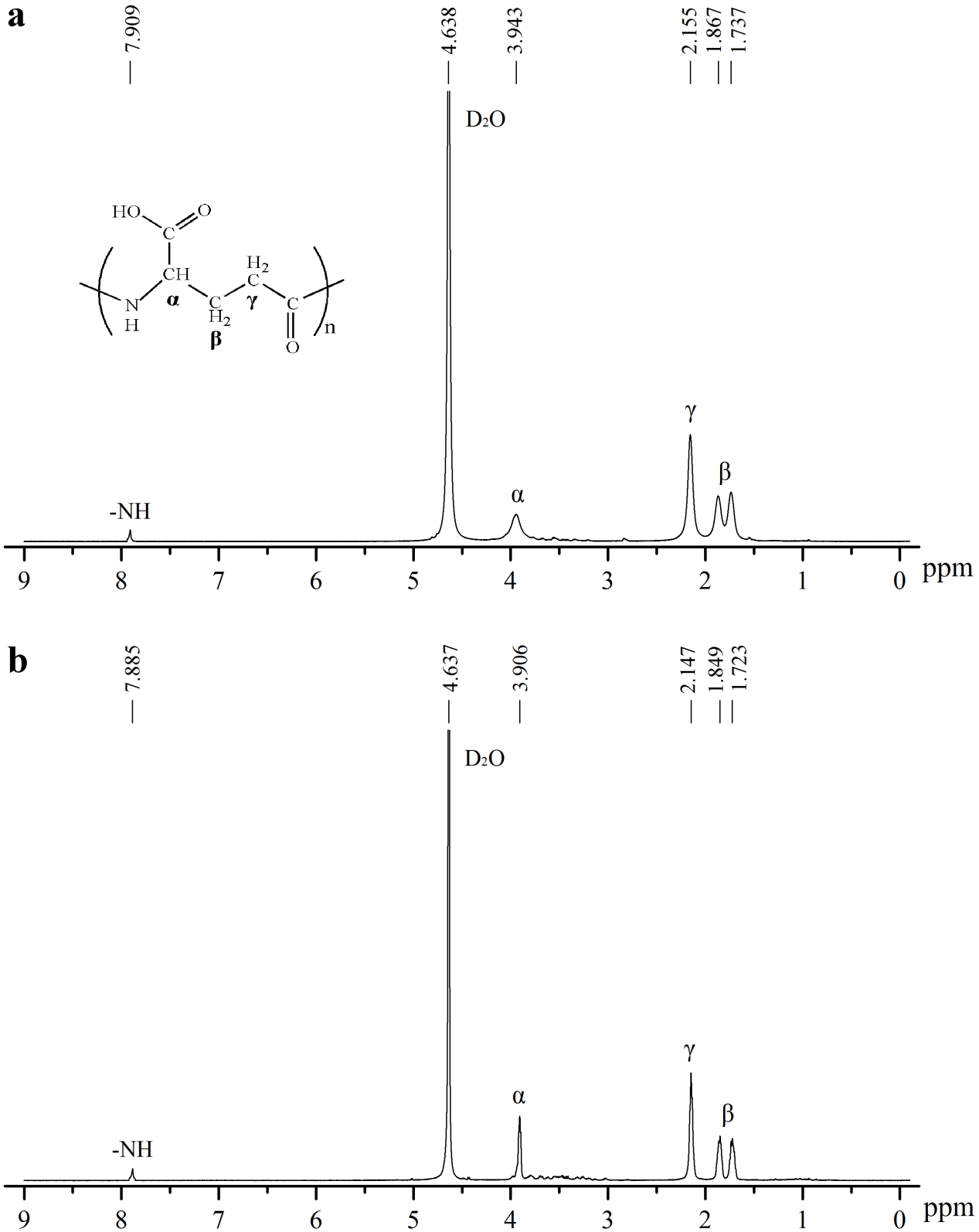
**

**Fig. S3.** The Venn diagram of SNPs, InDels, SVs and mutated genes between GXA-28 and GXG-5. a, SNPs; b, InDels; c, SVs; d, mutated genes.
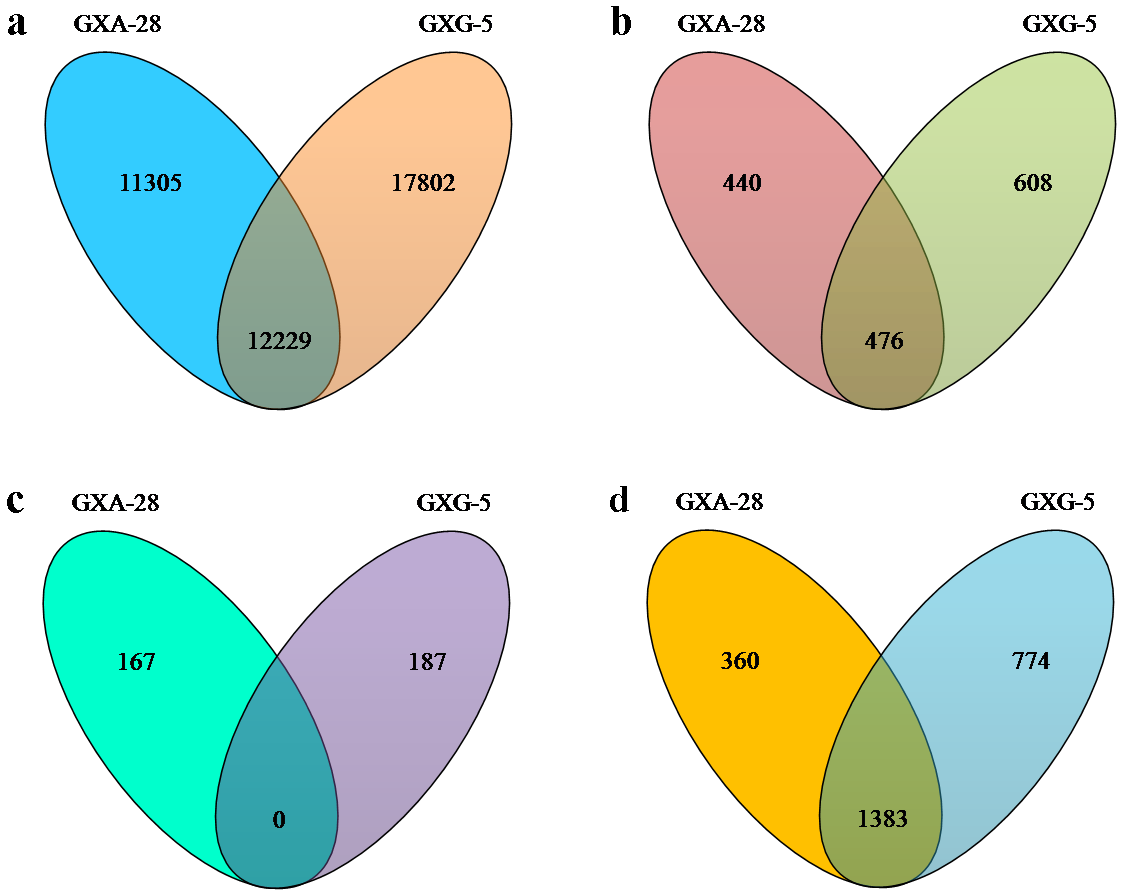


**Fig. S4.** A proposed pathway and regulatory system of γ-PGA synthesis in *B. subtilis*.


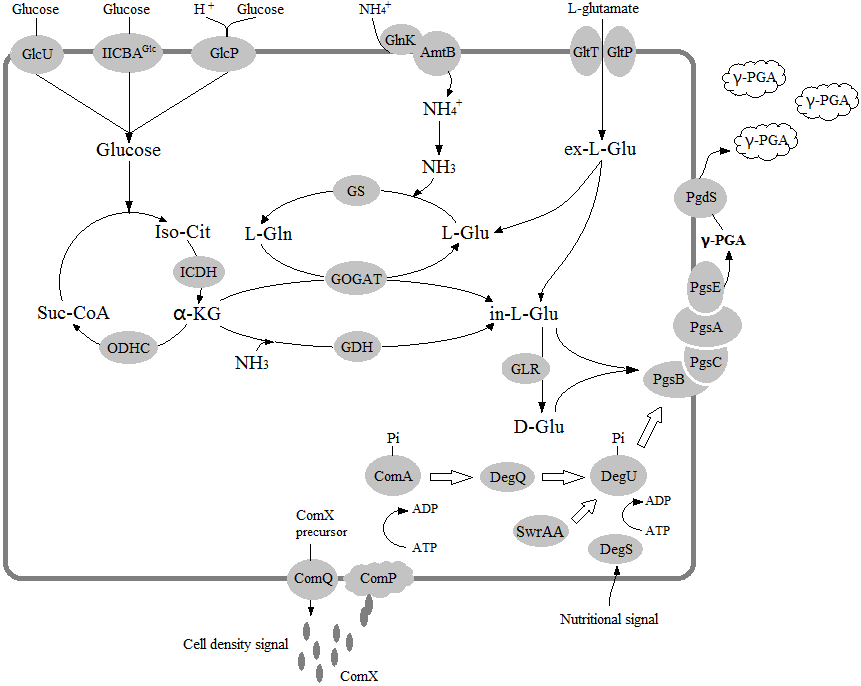


Gray ellipse represented enzyme or protein. Abbreviations: GlcU, glucose uptake protein, encoded by *glc*U; IICBA^Glc^, glucose-specific PTS permease, encoded by *pts*G, *pts*H, *pts*I and *glc*T; GlcP, hexose/H^+^ symporter, encoded by *glc*P; GlnK, nitrogen regulatory PII-like protein, encoded by *nrg*B; AmtB, ammonium transporter, encoded by *nrg*A; GltT, glutamate symport protein, encoded by *glt*T; GltP, glutamate symport protein, encoded by *glt*P; ICDH, isocitrate dehydrogenase, encoded by *icd*; ODHC, 2-oxoglutarate dehydrogenase E1 component, encoded by *odh*A; GS, glutamine synthetase, encoded by *gln*A; GOGAT, glutamate synthase, encoded by *glt*A and *glt*B; GDH, glutamate dehydrogenase, encoded by *gud*B or *roc*G; GLR, glutamate racemase, encoded by *yrp*C or *rac*E; PgsB, γ-PGA synthetase component, encoded by *pgs*B; PgsC, γ-PGA synthetase component, encoded by *pgs*C; PgsA, γ-PGA synthetase component, encoded by *pgs*A; PgsE, γ-PGA synthetase component, encoded by *pgs*E; PgdS, γ-PGA degradase, encoded by *pgd*S; ComQ, competence regulatory protein, encoded by *com*Q; ComX, *Bacillus* competence pheromone, encoded by *com*X; ComP, sensor histidine kinase, encoded by *com*P; ComA, transcriptional regulatory protein, encoded by *com*A; DegQ, degradation enzyme regulation protein, encoded by *deg*Q; DegS, sensor histidine kinase, encoded by *deg*S; DegU, transcriptional regulatory protein, encoded by *deg*U; SwrAA, swarming motility protein, encoded by *swr*AA.

Iso-Cit, isocitric acid; α-KG, α-ketoglutarate; Suc-CoA, succinyl coenzyme A; L-Gln, L-glutamine; L-Glu, L-glutamate; in-L-Glu, endogenous L-glutamate; ex-L-Glu, exogenous L-glutamate; D-Glu, D-glutamate.

**Table S1** Morphological and physiological characteristics of GXG-5.

| Items | GXG-5 | Items | GXG-5 |
| --- | --- | --- | --- |
| Cell form | rod | Gas from glucose | − |
| Cell size | 0.5-0.7 × 1.0-3.0 μm | Urease production | + |
| Spores | + ^a^ | Indole formation | − |
| Motility | + | Phenylalanine deaminase | − |
| Gram stain | + | Ammonium salts | + |
| Catalase | + | Nitrate reduction | + |
| Lecithase | + | Utilization of |  |
| Tyrosine hydrolase | + | D-glucose | + |
| VP test | + | D-Fructose | + |
| Anaerobic growth | − ^b^ | D-Sucrose | + |
| Growth |  | D-lactose | + |
| 37 ^◦^C | + | D-maltose | + |
| 50 ^◦^C | + | D-mannitol | + |
| pH 5.0 | + | Citrate | − |
| pH 9.0 | + | Hydrolysis of |  |
| NaCl 5% | + | Starch | + |
| NaCl 10% | + | Casein | + |
| NaCl 12% | − | Gelatin | + |

^a^ positive, ^b^ negative.

**Table S2** Statistics of resequencing results.

| Statistics | GXA-28 | GXG-5 |
| --- | --- | --- |
| Genome size (Mb) | 4.07 | 4.07 |
| Raw reads | 3920942 | 4728904 |
| Clean reads | 3919388 | 4727445 |
| Mapped (%) | 96.01 | 95.15 |
| Average depthe of genome | 281 | 341 |
| Coverage of genome 10× (%) | 96.21 | 92.72 |
| GC % | 44.3 | 43.98 |
| Q20 (%) | 95.71 | 95.49 |
| Q30 (%) | 89.73 | 89.37 |

**Table S3** Summary of SNPs from GXA-28 and GXG-5 against reference genome.

| SNPs | Categories | GXA-28 | GXG-5 |
| --- | --- | --- | --- |
| Total |  | 23534 | 30031 |
| Types | Transition | 16320 | 20824 |
|  | Transversion | 7214 | 9207 |
|  | Ti/Tv | 2.26 | 2.26 |
| Homo/Hete | Homozygosity | 23444 | 29830 |
|  | Heterozygosity | 90 | 201 |
|  | Het-Ratio (%) | 0.38 | 0.67 |
| Region | CDS | 19632 | 24886 |
|  | Upstream | 1978 | 2514 |
|  | Downstream | 1923 | 2621 |
|  | Intragenic | 1 | 1 |
|  | Intergenic | 0 | 9 |
| Function | Start Lost | 7 | 8 |
|  | Non Synonymous Start | 1 | 2 |
|  | Synonymous Coding | 15288 | 19347 |
|  | Non Synonymous Coding | 4311 | 5494 |
|  | Synonymous Stop | 13 | 21 |
|  | Stop Gained | 5 | 7 |
|  | Stop Lost | 7 | 7 |

**Table S4** Summary of InDels from GXA-28 and GXG-5 against reference genome.

| InDels | Categories | GXA-28 | GXG-5 |
| --- | --- | --- | --- |
| Total | - | 916 | 1084 |
| Region | CDS | 197 | 232 |
|  | Upstream | 311 | 358 |
|  | Downstream | 402 | 482 |
|  | Intragenic | 1 | 1 |
|  | Intergenic | 4 | 9 |
|  | Other | 1 | 2 |
| Function | Start Lost | 5 | 4 |
|  | Frame Shift | 128 | 147 |
|  | Codon Deletion | 17 | 22 |
|  | Codon Insertion | 19 | 19 |
|  | Codon Change Plus Codon Deletion | 10 | 15 |
|  | Codon Change Plus Codon Insertion | 9 | 13 |
|  | Stop Gained | 3 | 2 |
|  | Stop Lost | 6 | 10 |

**Table S5** Summary of SVs from GXA-28 and GXG-5 against reference genome.

| SVs | Categories | GXA-28 | GXG-5 |
| --- | --- | --- | --- |
| Total |  | 167 | 187 |
| Types | INS | 103 | 115 |
|  | DEL | 31 | 33 |
|  | INV | 22 | 26 |
|  | ITX | 10 | 13 |
|  | CTX | 0 | 0 |
|  | UN | 1 | 0 |

**Table S6** Summary of variant genes from GXA-28 and GXG-5 against reference genome.

| Variant genes | GXA-28 | GXG-5 |
| --- | --- | --- |
| Total | 1743 | 2157 |
| Genes with Non-synonymous SNP | 1637 | 2057 |
| Genes with InDel | 163 | 189 |
| Genes with SV | 144 | 146 |

**Table S7** Variations in the related genes of γ-PGA synthesis.

| **Process** | **Gene** | **Locus tag** | **Protein** | **Position** | **GXA-28** | **GXG-5** | **Effect** |
| --- | --- | --- | --- | --- | --- | --- | --- |
| Glucose transport | *glc*T | BSGX _1543 | GlcT | 1456952 | T | G | Non_Synonymous_Coding |
|  | *pts*G | BSGX _1544 | PtsG | 1458856 | C | G | Non_Synonymous_Coding |
|  | *pts*I | BSGX _1546 | EI | 1460351 | T | A | Non_Synonymous_Coding |
|  | *glc*P | BSGX _1175 | GlcP | 1125179 | C | T | Non_Synonymous_Coding |
|  |  |  |  | 1125425 | C | T | Non_Synonymous_Coding |
|  |  |  |  | 1125749 | G | A | Non_Synonymous_Coding |
|  |  |  |  | 1126087 | G | A | Non_Synonymous_Coding |
|  | *glc*U | BSGX _449 | GlcU | 444644 | A | G | Non_Synonymous_Coding |
| Ammonium transport | *nrg*A | BSGX _3936 | AmtB | 3757903 | A | T | Non_Synonymous_Coding |
| Glutamate synthesis | *glt*B | BSGX _2020 | GOGAT | 2010008 | A | C | Non_Synonymous_Coding |
|  |  |  |  | 2009576 … 2009569 | - | 285 bp | Insertion |
|  | *glt*A | BSGX _2021 | GOGAT | 2010189 | G | C | Non_Synonymous_Coding |
|  |  |  |  | 2011146 | T | C | Non_Synonymous_Coding |
|  |  |  |  | 2012177 | C | T | Non_Synonymous_Coding |
|  |  |  |  | 2013684 | G | A | Non_Synonymous_Coding |
|  |  |  |  | 2014169 | T | C | Non_Synonymous_Coding |
|  |  |  |  | 2014523 | G | T | Non_Synonymous_Coding |
|  | *gud*B | BSGX _2502 | GDH | 2403053 | GCGCCTTCAC | G | Codon_Deletion |
|  | *roc*G | BSGX _4068 | GDH | 3880870 | C | T | Non_Synonymous_Coding |
|  | *yrp*C | BSGX _2902 | YrpC | 2738074 … 2739312 | - | 1271 bp | Deletion |
|  | *rac*E | BSGX _3072 | RacE | 2903224 | T | C | Non_Synonymous_Coding |
| γ-PGA synthesis | *pgs*E | BSGX _3871 | PgsE | 3697652 | T | C | Non_Synonymous_Coding |

GlcT, the PtsGHI operon antiterminator, consists of three domains: an RNA-binding domain and two PTS regulation domains (PRD-I, PRD-II). PtsG, PTS system glucose-specific IICBA component. EI, PTS system glucose-specific IICBA component. GlcP, hexose/H^+^ symporter. GlcU, glucose uptake protein. AmtB, ammonium transporter. GOGAT, glutamate synthase, *glt*B encoded the glutamate synthase [NADPH] small chain, *glt*A encoded the glutamate synthase [NADPH] large chain. GDH, glutamate dehydrogenase. YrpC and RacE, glutamate racemase. PgsE, γ-PGA synthase component.
